# COVID-19, emergency remote teaching evaluation: the case of Indonesia

**DOI:** 10.1007/s10639-021-10680-3

**Published:** 2021-08-12

**Authors:** Ani Cahyadi, Hendryadi, Sri Widyastuti, Suryani

**Affiliations:** 1 Universitas Islam Negeri Antasari, Banjarmasin, Indonesia; 2 Sekolah Tinggi Ilmu Ekonomi Indonesia Jakarta, Jakarta, Indonesia; 3 Universitas Pancasila, Jakarta, Indonesia; 4 Universitas Islam Negeri Sultan Maulana Hasanuddin Banten, Banten, Indonesia

**Keywords:** Emergency remote teaching, COVID-19, CIPP, Indonesia

## Abstract

The global crisis caused by the COVID-19 pandemic has challenged educational institutions worldwide to rapidly shift to an online mode of teaching. In this paper, we discuss the concept of emergency remote teaching (ERT), including its implementation and evaluation, in the context of higher education in Indonesia. The Context, Input, Process, and Product framework was used to evaluate the implementation of ERT based on the experiences of 45 faculty members and 82 students from seven universities and colleges in three provinces in Indonesia. This study revealed several points of view. First, the shift to the ERT process depends on various aspects: internal organizational resources (curriculum, staff development, and technology), and external challenges (lack of access to a fast, affordable, and reliable Internet connection and the socioeconomic problems of the participants). Second, the ERT learning design needs to be framed using three principles: simplicity, flexibility, and empathy. The schools/administrators understand that this is not a normal situation in which learning competency standards must be rigorously met. In a crisis, given the facts that show disparities in technology and Internet networks, curriculum fulfillment is not the sole issue; it is also important to care for and support learners during this difficult time. This study provides recommendations that will serve as input for future strategies and educational policies in Indonesia, and developing countries in general. Additionally, this study can also be used as a benchmark for evaluating learning in similar situations in other countries.

## Introduction

**Fig. 1:**
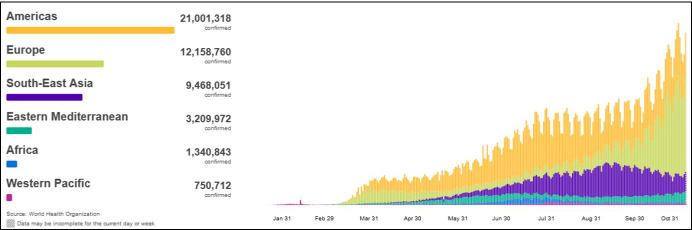
Confirmed cases across the WHO regions worldwide (WHO, 2020)

The spread of the deadly infectious disease, the novel coronavirus (also known as COVID-19), was declared a pandemic by the World Health Organization (WHO) on March 11, 2020 (Cucinotta & Vanelli, 2020). Based on data from the WHO Coronavirus Disease Dashboard (https://covid19.who.int) on November 5, 2020, it was claimed that COVID-19 had spread to more than 210 countries, with over 47,930,397 confirmed cases and 1,221,781 deaths (a 2.55% mortality rate). Figure 1 illustrates the graphical distribution of COVID-19 worldwide, indicating that the highest number of confirmed cases had been reported in the Americas, followed by Europe and Southeast Asia. In Southeast Asia, India recorded the highest number of cases (8.3 million confirmed cases), followed by Indonesia with 421,731 cases, and Bangladesh with 414,164. Due to uncertainty regarding the end of the pandemic and the absence of specific vaccines or treatments of COVID-19 (Sohrabi et al., 2020), organizations around the world have begun to explore contingency plans to cope with the pandemic (Mohmmed et al., 2020).

The first two cases of COVID-19 in Indonesia were confirmed in Jakarta. At a press conference in Jakarta on March 2, 2020, President Joko Widodo announced a national epidemic and ordered large-scale social distancing to prevent the spread of COVID-19 (Gorbiano, 2020), including within the education sector. In line with the presidential instruction, the Minister of Education and Culture canceled national exams for all levels (elementary, junior, and high schools) on March 24, 2020 (Ghaliya, 2020). Furthermore, since March 2020, all higher education institutions in Indonesia were instructed to start preparing to implement online learning modes.

Indonesia has 4232 universities and colleges across 34 provinces. Of these, 95% are private universities, and only 5% are public universities. Regarding the readiness of universities to implement distance learning, the Minister of Research, Technology, and Higher Education, Mohamad Nasir, explained in 2019 that only 15–20 universities in Indonesia are e-learning ready. Similar information has also been provided by the Association of Private Higher Education (APTISI), that is, only 30% of private universities are capable of conducting distance learning via the Internet or online (CNN Indonesia, 2020). This shows that before the pandemic, the majority of universities in Indonesia were not ready to carry out distance learning.

The choice of implementing emergency education has become an obligation to maintain the continuity of education in Indonesia through emergency remote teaching (ERT) during the COVID-19 pandemic. ERT is defined as a sudden interim shift in learning delivery from the face-to-face to the online delivery mode in response to a disaster/crisis; in contrast, online learning involves the voluntary planning and design of virtual delivery (Hodges et al., 2020). To reiterate, ERT is a teaching solution amid the COVID-19 crisis and should not be classified as general online learning. The main purpose of ERT is not to ultimately convert conventional methods to e-learning, but rather to provide temporary access using media or platforms available and reliable during an emergency. Thus, the ERT method can be understood as a quick solution and should be distinct from “online learning” (Hodges et al., 2020). An interesting question from this situation is how the government, universities, and teachers deal with this situation quickly.

This study aims to evaluate the implementation of ERT and explore critical issues during the COVID-19 pandemic in Indonesian higher education. Given that Indonesia is vulnerable to natural disasters, we offer a more positive perspective in terms of ERT as an educational technology innovation under disaster/crisis circumstances. Our work uses qualitative data from interviews and questionnaire responses by faculty members to record their experiences, beliefs, course disruptions, challenges encountered, and institutional policies on ERT implementation during the early semester (July 2020) to the end of the semester (January 2021). The Context, Input, Process, and Product (CIPP) model approach is recommended by Hodges et al. (2020) to provide comprehensive information regarding the implementation of learning during the pandemic.

This study contributes to the literature through practical experiences regarding teaching during crises in Indonesia. Online learning requires a stable and high-speed Internet connection. However, many areas in Indonesia are not part of a high-speed Internet network, which creates an obstacle to online learning. Thus, this study provides ERT practice from a different perspective from that of previous studies conducted in developed countries. Still, we are also aware that the prevalence of observed or experienced phenomena may differ between universities and other regions. Differences in the severity of the spread of the disease, readiness of technology and resources, socio-economics, and government policies causes academic institutions and universities in various countries respond in diverse ways. However, our study, conclusions, and recommendations can be used to compare similar issues in other countries.

## Overview of study design and participants

We adopted a multi-case methodology for this study. Specifically, it focuses on the experiences of faculty and teachers in Indonesia to overcome learning challenges during crises. The data were collected from interviews and open questionnaire responses by faculty members to record their experiences, beliefs, course disruptions, challenges, and institutional policies on the ERT.

We selected 45 participants, consisting of the Dean, Associate Dean of Academics, faculty quality assurance (55%), and faculty staff (45%) from seven universities and colleges in three provinces in Indonesia. The heads of each university approved participation through oral communication, following which the participants were required to voluntarily fill out the open questionnaire in an online survey. In this emergency situation, no formal ethics approval was provided for data collection. The data collected from both administrators and lecturers were regarded as evaluative rather than for research purposes, and the primary goal was to evaluate the implementation of remote teaching, which is ongoing. This study uses an informal and collegial approach by a survey coordinator (in this study as a co-author), Prof. Sri Widyastuti.

The CIPP model approach recommended by Hodges et al. (2020) was used to evaluate the implementation of ERT during a pandemic. The study was conducted in four phases. In the firstphase, faculty members responded to a written survey consisting of open questions that addressed the needs, problems, opportunities, challenges, and affordances based on individual considerations (Stufebeam & Coryn, 2014). In the second phase, input evaluation aimed to explore information about the program’s strategy, action plan, staffing arrangements, and budget for feasibility and potential cost-effectiveness to meet targeted needs and achieve goals (Hodges et al., 2020). Phases 1 and 2 involved 21 participants consisting of the Dean, Associate Dean of Academics, and faculty quality assurance from seven different universities.

The third phase required a series of monitoring activities, documents, and reports on the implementation of plans. This phase involved 24 senior lecturers from the same university in Phases 1 and 2. In the third phase, participants joined an online video-based structured focus group (Creswell & Creswell, 2017).

The fourth phase refers to the outcomes of the ERT initiative, including quantitative results, course completion rates, student attendance rates, and feedback provided by faculty for future requirements (Hodges et al., 2020). Additionally, quantitative data were obtained from 82 students, and 45 faculty members who responded on a 4-point Likert scale ranging from “excellent = 4” to “poor = 1” on the three questions covering the three ERT principles: simplicity, flexibility, and empathy (University of Auckland, 2020). Data from this stage were analyzed using descriptive statistical analysis.

## Results and discussion

The global COVID-19 outbreak has pushed many universities in the world to transform their conventional classroom course learning into online versions in an astonishingly short period. The global crisis has fundamentally changed the course delivery mode, but has also changed the methods by which teaching delivery and assessment can occur. Experience from the early phase of the COVID-19 pandemic (March–June 2020) provided valuable input for administrators and teachers in implementing ERT in the “new adaptation” phase of learning (starting July 2020). The CIPP framework (Table 1) has four evaluation steps: context, input, process, and product. These steps are progressive and carried out at different times: Steps 1 and 2 (beginning semester), Step 3 (middle of the semester), and Step 4 (end of the semester).

**Table 1:** CIPP evaluation

Phase 1	Phase 2	Phase 3	Phase 4
New semester (July, 2020)	New semester (July, 2020)	Mid semester (October, 2020)	End Semester (January, 2021)
Context	Input	Process	Product
Assessment of needs, goals, problems, and opportunities from internal and external resources and situations.	Assessment of the program’s strategy, action plan, staffing arrangements, and budget for feasibility and potential cost-effectiveness.	Assessment of monitoring activities, documents, and report on the implementation of plans.	Assessment of the outcomes of the ERT initiative.

Source: Adapted from Stufflebeam and Stufebeam and Coryn (2014), Stufflebeam and Zhang (2017), Hodges et al. (2020)

### Context evaluation

Based on our focus group discussion, several difficulties were observed during the execution of the ERT model based on the experience of the previous semester. These difficulties can be attributed to the following factors:

- The majority of universities do not have a learning management system (LMS).
- There were difficulties in determining which online platforms could be accessed by students and teachers.
- The lack of access to a fast, affordable, and reliable Internet connection in some areas of Indonesia is a fundamental problem in implementing ERT, and this problem has been highlighted by previous researchers (Mohmmed et al., 2020; Reich et al., 2020).
- Frequent technical problems that cause difficulties for students in meeting deadlines for completing assignments and exams.
- Inequality of devices used: The majority of students use cell phones as learning devices.

The results were based on open-ended questionnaires, which illustrate various internal and external challenges. We asked two additional questions about the understanding of ERT in terms of the learning system: “Does your university have an LMS?” More than 80% of the respondents said that their universities had this system. Next, we asked, “Does your university have an integrated learning system that has a variety of tools for teaching (video recording, online classes, etc.), assignments and examinations, and a student evaluation/assessment?” More than 90% answered that they did not have the system. The following is a comment from the teaching staff.

“We use various applications such as WhatsApp, Google Classroom, Google Meet, and Zoom. For student assessment, we have SIAKAD (Academic Information System).”

Unlike the initial phase of school closure (March 2020), administrators and teachers were more ready at the beginning of the semester in July 2020. With all these hurdles, the adopted ERT encompasses several opportunities and challenges for both faculty and teachers.

- Teachers become familiar with various online learning platforms.
- The teacher already understands that ERT is a temporary solution, so that the implementation of learning promotes the principles of simplicity, flexibility, and empathy.
- Teachers become more creative in developing online-based learning media, such as video recordings, online exams, and other supporting documents.
- Transformation from conventional teaching to online teaching bridges the skill gap between teachers, especially in applying recent advanced learning technologies.

### Input evaluation

Input evaluation to provide information about the sources can be used to implement ERT during COVID-19. The basic requirements were classified into four indicators: (1) Was the technology infrastructure sufficient to handle the needs of ERT? (2) Did the campus support staff have sufficient capacity to handle the needs of ERT? (3) How was the readiness of learning resources (such as devices, modules, and learning guidelines)? (4) Learning delivery (such as available media to carry out ERT, Google Meet, Zoom, and Google Classroom).

This study found that the majority of respondents stated “Internet speed instability” as a major issue for lecturers and students. Applications such as WhatsApp, Google Classroom, Google Meet, and Zoom were used in combination as the learning delivery media by the majority of respondents. These applications were chosen based on considerations of accessibility, convenience, and in general, ease of use, both by lecturers and students. Accordingly, we agree that the learning delivery used met the elements of accessibility and lecturers creatively innovate in delivering material by combining video recordings, modules, and power points. One respondent argued:

“We conducted an initial discussion through the WhatsApp group to determine what application was the most suitable and easily accessible to students. Finally, from the discussion, the applications for the most efficient and accessible virtual class were Gmeet and Zoom, while for our assignments and discussions, weuse a mix of Google Classroom and WhatsApp Group...”

ERT is focused on delivering practical learning with quick and simple approaches to the online delivery of materials and assignments. ERT is not intended to meet learning objectives and standards in normal times, but rather to provide convenience by reducing basic competencies and study subjects. Thus, ERT is neither an attempt to fully teach the subjects in an online mode using various “advanced” applications, nor is it the time to strive for the “best practice” in online delivery (Wang & East, 2020). In the present situation, the majority of campuses have support teams that are available to help faculty members implement online teaching even though the team members are limited in number and capacity. In some situations, the support team also helps lecturers upload teaching materials and create virtual classes. One lecturer argued the following:

“The support team is relatively limited, but we still appreciate what they have done, especially for lecturers who do not have the experience of doing online learning. The initial implementation is very chaotic, but over time, lecturers can learn to get used to teaching online through various available platforms.”

In general, the responses on various adopted tools, including Gmeet, Zoom, Google Classroom, and WhatsApp Group, are deemed to be the most efficient and accessible for teachers and students. However, there ares sometimes technical problems, especially in areas where the Internet connection is low and unstable.

Another issue faced in implementing ERTs is related to the curriculum. In general, respondents stated that the implementation of ERT still uses the standard curriculum due to delays in implementing an emergency curriculum from the Indonesian Ministry of Education and Culture. The emergency curriculum (under special conditions) prepared by the Ministry is basically a simplification of the national curriculum. In the emergency curriculum, basic competencies are reduced for each subject, so that teachers and students can focus on essential and prerequisite competencies for continuing learning at the next level.

### Process evaluation

Process evaluation requires a series of monitoring activities, documents, and reports on the implementation of plans. Process evaluation identifies several points of concern from respondents:

- More than 70% of the respondents answered that the faculty monitors learning activities to achieve quality teaching and learning processes.
- Difficulty preventing and controlling fraudulent practices (e.g., plagiarism, claims of other people’s work, and cheating on exams).
- There are still frequent technical problems that cause difficulties for students in meeting deadlines for completing assignments and exams.
- Using special programs that require fast Internet access and high random-access memory (RAM) on mobile devices will cause new problems, such as obstruction of the learning process.

The pandemic crisis has changed the environment of society, education, the economy, and the individual. From the perspective of complexity theory, the systems are unpredictable, and organizations must be able to continue to interact and obtain accompanying feedback on what to do while considering social and organizational changes. Thus, the implementation of ERT needs to emphasize a shared responsibility among faculty members and supporting staff (Oliver & Hyun, 2011), and requires a collective decision from all participant groups (including students) rather than a centrally managed plan (Wang & East, 2020). A senior lecturer stated:

“We try to be flexible as possible to make it easy for students. We understand that the current situation is unfavorable, and many of us have also been directly affected by this pandemic. But we also keep our virtual meetings on schedule to monitor the student’s condition, I think this is the best way we can do…”

Using special programs that require fast Internet access and high RAM on mobile devices will cause new problems, such as obstruction of the learning process. Consequently, there are many technical problems that occur in video conferences, such as loss of sound, delayed images, or inability to access classes due to low Internet networks and the technical capabilities of the devices used (for example, devices do not meet the minimum requirements for the application).

### Product evaluation

Product evaluation refers to the outcomes of the ERT initiative: (1) quantitative outcome: course completion and student attendance rates; (2) form of faculty support in special cases (for example, students and teachers who were directly exposed to COVID-19), and (3) feedback provided by faculty and teachers for future ERT requirements (Hodges et al., 2020). We asked two questions about quantitative outcomes: course completion and student attendance rates. Although more than 50% of the respondents stated that material achievement and student interaction had gone well, over 65% preferred face-to-face learning in the post-pandemic period. A limitation of technology resources is that obstacles are encountered during online tutorials with low and unstable Internet networks in several regions of Indonesia. This emphasizes the fact that the application of distance learning in Indonesia still requires time and an in-depth evaluation before being widely implemented.

"Do not assume that students cannot complete assignments or do not respond to questions posed in online classes as lazy behavior. Students may not have stable Internet access, be in an unfavorable situation, share devices with other family members, or have no devices at all. "

Another effort made by the faculty was to provide information and a complaint center. Some universities provide a 15% discount on tuition fees, waivers for tuition fees, and even full scholarships. They also provide assistance with Internet quota fees and several other policies to ensure that students in poor financial situations can continue their studies. ERT focuses more on the teacher’s efforts to execute the learning function and is feasible for remote online delivery without enhancing stress among students and teachers during difficult times (Wang & East, 2020). A lecturer gave the following opinion:

“We are fully aware that many families have been directly affected by the pandemic, such as the inoperability of the business sector, reduced salaries, and even job cuts experienced by parents of students. I always give messages to lecturers to actively ask about the conditions of students, giving them enthusiasm if someone is hit by a bad situation, and continue to maintain student learning motivation. So far, I have heard that lecturers have a direct connection with student groups through WhatsApp groups, so that any information can be easily discovered by the lecturer.”

As shown in Table 2, the descriptive analysis results show that the overall rating score ranges from 2.61 to 3.03, with the highest rating for flexibility, followed by simplicity and empathy. Statistical analysis using nonparametric testing (Mann-Whitney U Test) found that there were significant differences between faculty and students on the principles of “simplicity,” “flexibility,” and “empathy” (*p* < 0.05). All principles were rated higher by faculty members than by students (see Table 2).

**Table 2:** Perceptions of ERT by faculty and students (poor to excellent)

	N	Group	Mean	*SD*	Mann-Whitney U Test	
					Z	Asymp. Sig.
Simplicity	82	S	2.35	1.011	−2.65	.01
	45	FM	2.92	.702		
	127	Overall	2.64			
Flexibility	82	S	2.37	.949	−5.48	.00
	45	FM	3.68	.627		
	127	Overall	3.03			
Empathy	82	S	2.13	.953	−4.44	.00
	45	FM	3.08	.702		
	127	Overall	2.61			

*S* students, *FM* Faculty members, *SD* Standard deviation; Faculty member: *n* = 45; Student: *n* = 82

Table 2 show differences in perceptions between students and faculty members in assessing ERT implementation throughout the semester. Sequentially, students gave the highest ratings to the principle of flexibility, followed by simplicity and empathy in implementing remote teaching. Conversely, faculty members provide the highest ratings to flexibility, followed by empathy and simplicity.

### Implications

Through the CIPP framework, we found that there were no serious problems faced by the faculty in implementing ERT. In terms of context and input, teachers and staff properly understand the various problems and what is needed to make plans at the beginning of the semester based on previous experiences. Unlike the initial phase of school closure (March 2020), administrators and teachers were ready at the beginning of the semester in July 2020, even though various technical obstacles were still found in implementing learning. The schools/administrators understand that this is not a normal situation in which learning competency standards must be rigorously met. In a crisis, given the facts that show disparities in technology and Internet networks, curriculum fulfillment is not the sole issue; it is also important to care for and support learners during this difficult time. The lack of access to a fast, affordable, and reliable Internet connection in some areas of Indonesia is a fundamental problem in implementing ERT. Without infrastructural support, the implementation of online education is not effective. The pandemic has made us aware that Indonesia still has significant limitations, especially in terms of reliability, stability, and low-cost Internet access.

#### Internal resources

The implemented ERT model in Indonesia’s higher education had great responses from teachers and students; however, several challenges should be highlighted and improved. First, the schools/administrators need to understand that this is not a normal situation in which learning competency standards must be rigorously met. Curriculum fulfillment is not the sole issue amidst disparities in technology and Internet networks, health anxiety, and economic problems resulting from the pandemic. Administrators should focus more on keeping students motivated by empathy, caring for, and supporting learners during this difficult time. Implementing ERT through the adoption of a standardized curriculum invalidates the goals of ERT, which is not intended to meet learning objectives and standards in normal times, but rather aims to provide flexibility and convenience by reducing basic competencies and study subjects. In the long term, the Indonesian government needs to develop an emergent curriculum that can accommodate the needs of areas that may be affected by major natural disasters.

Second, university administrators must ensure that teaching staff has two essential competencies: technical and pedagogical. To be effective, they should focus on lecturers’ technical skills in running ERT. Although this task can be assisted by IT support in some ways, for the effectiveness of future learning, each lecturer needs to be adequately equipped with technical knowledge and skills in managing online-based learning. Lecturers can take advantage of various free resources such as YouTube and OpenLearn, as well as other open education resources, to enrich the digital education delivery system.

Third, the principle of ERT is to provide learning activities that are simple, accessible, affordable, flexible, and provide clear support to students with an empathetic attitude rather than just delivering the best lectures (Bozkurt & Sharma, 2020). Apart from technical skills, pedagogical ability in managing learning is the most important aspect. This ability is needed to maintain student motivation in difficult situations. However, the three principles of simplicity, flexibility, and empathy (University of Auckland, 2020) will not be realized if school administrators continue to implement the standard curriculum during the COVID-19 pandemic.

Fourth, the evaluation results show that efforts are needed to prevent and control fraudulent practices in ERT (e.g., plagiarism, claims of other people’s work, and cheating on exams). Plagiarism in the online environment has become a concern in the last decade because of the increasing use of technology in education (Denney et al., 2021; Ison, 2014). Administrators and educators need to apply a percentage of students’ final scores in the learning process compared to written exams. In other words, a larger portion of the assessment can be directed at group task activities and online discussions. The use of written exams should be minimized, especially in certain subjects with high theoretical content. If written exams cannot be avoided, Turnitin and Plagiarism Check can be an option to detect the level of similarity of students’ test results.

#### External resources

The lack of access to a fast, affordable, and reliable Internet connection in some areas of Indonesia is a fundamental problem in implementing ERT. The final evaluation results noted that many students had difficulty participating in online learning because of the instability of the Internet speed and the compatibility issues of their devices with applications used by teachers. Consequently, many technical problems have occurred, such as loss of sound, delayed images, and inability to access classes. Without adequate technological support from administrators to students, the implementation of online education will not be effective. Thus, administrators and teachers need to conduct socialization and online learning trials for students before the semester starts to ensure that the application can be accessed properly when the term begins.

ERT implementation cannot be separated from the psychological and socioeconomic aspects. Administrators and teachers cannot obsessively focus on teaching delivery, knowledge transmission, and lecturing using sophisticated technology (Bozkurt & Sharma, 2020). The application of learning technology and the need to consider broad accessibility aspects and flexible learning modes also need to pay attention to aspects regarding affordability. In other words, administrators and teachers should consider whether students can afford to pay fees related to online learning. For example, using a virtual class/videoconference in an asynchronous mode, apart from requiring fast Internet access, can also consume large volumes of Internet data. Most Indonesian Internet users rely on expensive limited-capacity mobile networks (Harto, 2020), which makes it difficult for students to use broadband networks to meet their online learning requirements. We appreciate the work of lecturers who have a sense of social responsibility not to impose specific applications as mediums for delivering the material. The majority of teachers stated that specific applications need to be discussed with students to ensure that the learning process can run efficiently. Thus, learning delivery can use a mixture of synchronous and asynchronous environments based on an evaluation of the situation.

### Limitations and future research directions

The present study has several limitations. First, this study is a case study aimed at evaluating ERT implementation in educational institutions, especially in Indonesia. This study culminates in a comprehensive understanding of the administrators and teachers, the area under investigation, and ideally for policy improvement in the future. Second, this study uses a case study approach with a convenience sampling method, which also leads to some restrictions regarding generalization.

We argue that this approach is well-suited for this study for two reasons. First, data collection in crisis contexts can be highly unstructured and unpredictable (Lin et al., 2017). This situation allowed us to acknowledge the novelty of the ERT phenomenon quickly compared to gathering extensive data that would take a long time to validate the theories. Second, this study uses a qualitative approach, where the focus is on the subjects’ meaning, using various data collection techniques and analytical procedures. According to Saunders et al. (2012), data collection in qualitative research is not standardized; questions and procedures can arise and change during a naturalistic and interactive research process. Research success is entirely based on the researcher’s ability to gain physical access to resources, build relationships, and demonstrate sensitivity to gain cognitive access to data sources (Saunders et al., 2012). Based on these limitations, we invite future researchers to evaluate ERT implementation in different situations and countries. Furthermore, other models such as the ACAD framework (Carvalho & Goodyear, 2018) and the ADDIE model (Analyze, Design, Develop, Implement, and Evaluate) as a framework for designing and developing educational and training programs (Kurt, 2017).

## Final remarks

This study aims to evaluate the implementation of ERT in the adaptation phase, which started from July 2020 to January 2021. Through the CIPP framework, we found that there were no serious problems faced by the faculty in implementing ERT. In terms of context and input, teachers and staff properly understand the various problems and what is needed to make plans at the beginning of the semester based on previous experiences. Unlike the initial phase of school closure (March 2020), administrators and teachers were ready at the beginning of the semester in July 2020, even though some technical obstacles remain in the implementation of ERT. From a practical viewpoint, the ERT’s entire shifting process should be conducted considering various aspects: internal and external. Internal organizational resources such as curriculum, staff development, and technology resources are the three main components of ERT implementation. Meanwhile, the external challenges consist of a lack of access to a fast, affordable, and reliable Internet connection in some areas and the socioeconomic problems of the participants. Attention to internal and external aspects also needs to be framed using three principles: simplicity, flexibility, and empathy.

Finally, this study provides valuable insights for education practitioners, policymakers, and researchers to understand the current situation as a reflection of national educational technology readiness. In the long run, in Indonesia in particular, researchers and education practitioners will have to collaborate to design a national online curriculum that has the capacity to increase flexibility and convenience without sacrificing the quality of education. Online learning is a potential choice for the future, but its implementation needs to consider various aspects, namely infrastructure and technology resources (hardware, software, devices, and a fast, affordable, and reliable Internet connection), financial resources, and the organizational environment (curriculum, support technicians, teachers, materials, and others).

## Data Availability

Data sharing is not applicable to this article, as no new data were created or analyzed in this study.
